# COVID-19 pandemic and its impact on social relationships and health

**DOI:** 10.1136/jech-2021-216690

**Published:** 2021-08-19

**Authors:** Emily Long, Susan Patterson, Karen Maxwell, Carolyn Blake, Raquel Bosó Pérez, Ruth Lewis, Mark McCann, Julie Riddell, Kathryn Skivington, Rachel Wilson-Lowe, Kirstin R Mitchell

**Affiliations:** 1 MRC/CSO Social and Public Health Sciences Unit, University of Glasgow, Glasgow, UK; 2 MRC/CSO Social and Public Health Sciences Unit, Institute of Health & Wellbeing, University of Glasgow, Glasgow, UK

**Keywords:** COVID-19, health, inequalities

## Abstract

This essay examines key aspects of social relationships that were disrupted by the COVID-19 pandemic. It focuses explicitly on relational mechanisms of health and brings together theory and emerging evidence on the effects of the COVID-19 pandemic to make recommendations for future public health policy and recovery. We first provide an overview of the pandemic in the UK context, outlining the nature of the public health response. We then introduce four distinct domains of social relationships: social networks, social support, social interaction and intimacy, highlighting the mechanisms through which the pandemic and associated public health response drastically altered social interactions in each domain. Throughout the essay, the lens of health inequalities, and perspective of relationships as interconnecting elements in a broader system, is used to explore the varying impact of these disruptions. The essay concludes by providing recommendations for longer term recovery ensuring that the social relational cost of COVID-19 is adequately considered in efforts to rebuild.

## Introduction

Infectious disease pandemics, including SARS and COVID-19, demand intrapersonal behaviour change and present highly complex challenges for public health.^1^ A pandemic of an airborne infection, spread easily through social contact, assails human relationships by drastically altering the ways through which humans interact. In this essay, we draw on theories of social relationships to examine specific ways in which relational mechanisms key to health and well-being were disrupted by the COVID-19 pandemic. Relational mechanisms refer to the processes between people that lead to change in health outcomes.

At the time of writing, the future surrounding COVID-19 was uncertain. Vaccine programmes were being rolled out in countries that could afford them, but new and more contagious variants of the virus were also being discovered. The recovery journey looked long, with continued disruption to social relationships. The social cost of COVID-19 was only just beginning to emerge, but the mental health impact was already considerable,^2 3^ and the inequality of the health burden stark.^4^ Knowledge of the epidemiology of COVID-19 accrued rapidly, but evidence of the most effective policy responses remained uncertain.

The initial response to COVID-19 in the UK was reactive and aimed at reducing mortality, with little time to consider the social implications, including for interpersonal and community relationships. The terminology of ‘social distancing’ quickly became entrenched both in public and policy discourse. This equation of physical distance with social distance was regrettable, since only physical proximity causes viral transmission, whereas many forms of *social* proximity (eg, conversations while walking outdoors) are minimal risk, and are crucial to maintaining relationships supportive of health and well-being.

The aim of this essay is to explore four key relational mechanisms that were impacted by the pandemic and associated restrictions: social networks, social support, social interaction and intimacy. We use relational theories and emerging research on the effects of the COVID-19 pandemic response to make three key recommendations: one regarding public health responses; and two regarding social recovery. Our understanding of these mechanisms stems from a ‘systems’ perspective which casts social relationships as interdependent elements within a connected whole.^5^

### Social networks

Social networks characterise the individuals and social connections that compose a system (such as a workplace, community or society). Social relationships range from spouses and partners, to coworkers, friends and acquaintances. They vary across many dimensions, including, for example, frequency of contact and emotional closeness. Social networks can be understood both in terms of the individuals and relationships that compose the network, as well as the overall network structure (eg, how many of your friends know each other).

Social networks show a tendency towards homophily, or a phenomenon of associating with individuals who are similar to self.^6^ This is particularly true for ‘core’ network ties (eg, close friends), while more distant, sometimes called ‘weak’ ties tend to show more diversity. During the height of COVID-19 restrictions, face-to-face interactions were often reduced to core network members, such as partners, family members or, potentially, live-in roommates; some ‘weak’ ties were lost, and interactions became more limited to those closest. Given that peripheral, weaker social ties provide a diversity of resources, opinions and support,^7^ COVID-19 likely resulted in networks that were smaller and more homogenous.

Such changes were not inevitable nor necessarily enduring, since social networks are also adaptive and responsive to change, in that a disruption to usual ways of interacting can be replaced by new ways of engaging (eg, Zoom). Yet, important inequalities exist, wherein networks and individual relationships within networks are not equally able to adapt to such changes. For example, individuals with a large number of newly established relationships (eg, university students) may have struggled to transfer these relationships online, resulting in lost contacts and a heightened risk of social isolation. This is consistent with research suggesting that young adults were the most likely to report a worsening of relationships during COVID-19, whereas older adults were the least likely to report a change.^8^

Lastly, social connections give rise to emergent properties of social systems,^9^ where a community-level phenomenon develops that cannot be attributed to any one member or portion of the network. For example, local area-based networks emerged due to geographic restrictions (eg, stay-at-home orders), resulting in increases in neighbourly support and local volunteering.^10^ In fact, research suggests that relationships with neighbours displayed the largest net gain in ratings of relationship quality compared with a range of relationship types (eg, partner, colleague, friend).^8^ Much of this was built from spontaneous individual interactions within local communities, which together contributed to the ‘community spirit’ that many experienced.^11^ COVID-19 restrictions thus impacted the personal social networks and the structure of the larger networks within the society.

### Social support

Social support, referring to the psychological and material resources provided through social interaction, is a critical mechanism through which social relationships benefit health. In fact, social support has been shown to be one of the most important resilience factors in the aftermath of stressful events.^12^ In the context of COVID-19, the usual ways in which individuals interact and obtain social support have been severely disrupted.

One such disruption has been to opportunities for spontaneous social interactions. For example, conversations with colleagues in a break room offer an opportunity for socialising beyond one’s core social network, and these peripheral conversations can provide a form of social support.^13 14^ A chance conversation may lead to advice helpful to coping with situations or seeking formal help. Thus, the absence of these spontaneous interactions may mean the reduction of indirect support-seeking opportunities. While direct support-seeking behaviour is more effective at eliciting support, it also requires significantly more effort and may be perceived as forceful and burdensome.^15^ The shift to homeworking and closure of community venues reduced the number of opportunities for these spontaneous interactions to occur, and has, second, focused them locally. Consequently, individuals whose core networks are located elsewhere, or who live in communities where spontaneous interaction is less likely, have less opportunity to benefit from spontaneous in-person supportive interactions.

However, alongside this disruption, new opportunities to interact and obtain social support have arisen. The surge in community social support during the initial lockdown mirrored that often seen in response to adverse events (eg, natural disasters^16^). COVID-19 restrictions that confined individuals to their local area also compelled them to focus their in-person efforts locally. Commentators on the initial lockdown in the UK remarked on extraordinary acts of generosity between individuals who belonged to the same community but were unknown to each other. However, research on adverse events also tells us that such community support is not necessarily maintained in the longer term.^16^

Meanwhile, online forms of social support are not bound by geography, thus enabling interactions and social support to be received from a wider network of people. Formal online social support spaces (eg, support groups) existed well before COVID-19, but have vastly increased since. While online interactions can increase perceived social support, it is unclear whether remote communication technologies provide an effective substitute from in-person interaction during periods of social distancing.^17 18^ It makes intuitive sense that the usefulness of online social support will vary by the type of support offered, degree of social interaction and ‘online communication skills’ of those taking part. Youth workers, for instance, have struggled to keep vulnerable youth engaged in online youth clubs,^19^ despite others finding a positive association between amount of digital technology used by individuals during lockdown and perceived social support.^20^ Other research has found that more frequent face-to-face contact and phone/video contact both related to lower levels of depression during the time period of March to August 2020, but the negative effect of a lack of contact was greater for those with higher levels of usual sociability.^21^ Relatedly, important inequalities in social support exist, such that individuals who occupy more socially disadvantaged positions in society (eg, low socioeconomic status, older people) tend to have less access to social support,^22^ potentially exacerbated by COVID-19.

### Social and interactional norms

Interactional norms are key relational mechanisms which build trust, belonging and identity within and across groups in a system. Individuals in groups and societies apply meaning by ‘approving, arranging and redefining’ symbols of interaction.^23^ A handshake, for instance, is a powerful symbol of trust and equality. Depending on context, not shaking hands may symbolise a failure to extend friendship, or a failure to reach agreement. The norms governing these symbols represent shared values and identity; and mutual understanding of these symbols enables individuals to achieve orderly interactions, establish supportive relationship accountability and connect socially.^24 25^

Physical distancing measures to contain the spread of COVID-19 radically altered these norms of interaction, particularly those used to convey trust, affinity, empathy and respect (eg, hugging, physical comforting).^26^ As epidemic waves rose and fell, the work to negotiate these norms required intense cognitive effort; previously taken-for-granted interactions were re-examined, factoring in current restriction levels, own and (assumed) others’ vulnerability and tolerance of risk. This created awkwardness, and uncertainty, for example, around how to bring closure to an in-person interaction or convey warmth. The instability in scripted ways of interacting created particular strain for individuals who already struggled to encode and decode interactions with others (eg, those who are deaf or have autism spectrum disorder); difficulties often intensified by mask wearing.^27^

Large social gatherings—for example, weddings, school assemblies, sporting events—also present key opportunities for affirming and assimilating interactional norms, building cohesion and shared identity and facilitating cooperation across social groups.^28^ Online ‘equivalents’ do not easily support ‘social-bonding’ activities such as singing and dancing, and rarely enable chance/spontaneous one-on-one conversations with peripheral/weaker network ties (see the Social networks section) which can help strengthen bonds across a larger network. The loss of large gatherings to celebrate rites of passage (eg, bar mitzvah, weddings) has additional relational costs since these events are performed by and for communities to reinforce belonging, and to assist in transitioning to new phases of life.^29^ The loss of interaction with diverse others via community and large group gatherings also reduces intergroup contact, which may then tend towards more prejudiced outgroup attitudes. While online interaction can go some way to mimicking these interaction norms, there are key differences. A sense of anonymity, and lack of in-person emotional cues, tends to support norms of polarisation and aggression in expressing differences of opinion online. And while online platforms have potential to provide intergroup contact, the tendency of much social media to form homogeneous ‘echo chambers’ can serve to further reduce intergroup contact.^30 31^

### Intimacy

Intimacy relates to the feeling of emotional connection and closeness with other human beings. Emotional connection, through romantic, friendship or familial relationships, fulfils a basic human need^32^ and strongly benefits health, including reduced stress levels, improved mental health, lowered blood pressure and reduced risk of heart disease.^32 33^ Intimacy can be fostered through familiarity, feeling understood and feeling accepted by close others.^34^

Intimacy via companionship and closeness is fundamental to mental well-being. Positively, the COVID-19 pandemic has offered opportunities for individuals to (re)connect and (re)strengthen close relationships within their household via quality time together, following closure of many usual external social activities. Research suggests that the first full UK lockdown period led to a net gain in the quality of steady relationships at a population level,^35^ but amplified existing inequalities in relationship quality.^35 36^ For some in single-person households, the absence of a companion became more conspicuous, leading to feelings of loneliness and lower mental well-being.^37 38^ Additional pandemic-related relational strain^39 40^ resulted, for some, in the initiation or intensification of domestic abuse.^41 42^

Physical touch is another key aspect of intimacy, a fundamental human need crucial in maintaining and developing intimacy within close relationships.^34^ Restrictions on social interactions severely restricted the number and range of people with whom physical affection was possible. The reduction in opportunity to give and receive affectionate physical touch was not experienced equally. Many of those living alone found themselves completely without physical contact for extended periods. The deprivation of physical touch is evidenced to take a heavy emotional toll.^43^ Even in future, once physical expressions of affection can resume, new levels of anxiety over germs may introduce hesitancy into previously fluent blending of physical and verbal intimate social connections.^44^

The pandemic also led to shifts in practices and norms around sexual relationship building and maintenance, as individuals adapted and sought alternative ways of enacting sexual intimacy. This too is important, given that intimate sexual activity has known benefits for health.^45 46^ Given that social restrictions hinged on reducing household mixing, possibilities for partnered sexual activity were primarily guided by living arrangements. While those in cohabiting relationships could potentially continue as before, those who were single or in non-cohabiting relationships generally had restricted opportunities to maintain their sexual relationships. Pornography consumption and digital partners were reported to increase since lockdown.^47^ However, online interactions are qualitatively different from in-person interactions and do not provide the same opportunities for physical intimacy.

## Recommendations and conclusions

In the sections above we have outlined the ways in which COVID-19 has impacted social relationships, showing how relational mechanisms key to health have been undermined. While some of the damage might well self-repair after the pandemic, there are opportunities inherent in deliberative efforts to build back in ways that facilitate greater resilience in social and community relationships. We conclude by making three recommendations: one regarding public health responses to the pandemic; and two regarding social recovery.

### Recommendation 1: explicitly count the relational cost of public health policies to control the pandemic

Effective handling of a pandemic recognises that social, economic and health concerns are intricately interwoven. It is clear that future research and policy attention must focus on the social consequences. As described above, policies which restrict physical mixing across households carry heavy and unequal relational costs. These include for individuals (eg, loss of intimate touch), dyads (eg, loss of warmth, comfort), networks (eg, restricted access to support) and communities (eg, loss of cohesion and identity). Such costs—and their unequal impact—should not be ignored in short-term efforts to control an epidemic. Some public health responses—restrictions on international holiday travel and highly efficient test and trace systems—have relatively small relational costs and should be prioritised. At a national level, an earlier move to proportionate restrictions, and investment in effective test and trace systems, may help prevent escalation of spread to the point where a national lockdown or tight restrictions became an inevitability. Where policies with relational costs are unavoidable, close attention should be paid to the unequal relational impact for those whose personal circumstances differ from normative assumptions of two adult families. This includes consideration of whether expectations are fair (eg, for those who live alone), whether restrictions on social events are equitable across age group, religious/ethnic groupings and social class, and also to ensure that the language promoted by such policies (eg, households; families) is not exclusionary.^48 49^ Forethought to unequal impacts on social relationships should thus be integral to the work of epidemic preparedness teams.

### Recommendation 2: intelligently balance online and offline ways of relating

A key ingredient for well-being is ‘getting together’ in a physical sense. This is fundamental to a human need for intimate touch, physical comfort, reinforcing interactional norms and providing practical support. Emerging evidence suggests that online ways of relating cannot simply replace physical interactions. But online interaction has many benefits and for some it offers connections that did not exist previously. In particular, online platforms provide new forms of support for those unable to access offline services because of mobility issues (eg, older people) or because they are geographically isolated from their support community (eg, lesbian, gay, bisexual, transgender and queer (LGBTQ) youth). Ultimately, multiple forms of online and offline social interactions are required to meet the needs of varying groups of people (eg, LGBTQ, older people). Future research and practice should aim to establish ways of using offline and online support in complementary and even synergistic ways, rather than veering between them as social restrictions expand and contract. Intelligent balancing of online and offline ways of relating also pertains to future policies on home and flexible working. A decision to switch to wholesale or obligatory homeworking should consider the risk to relational ‘group properties’ of the workplace community and their impact on employees’ well-being, focusing in particular on unequal impacts (eg, new vs established employees). Intelligent blending of online and in-person working is required to achieve flexibility while also nurturing supportive networks at work. Intelligent balance also implies strategies to build digital literacy and minimise digital exclusion, as well as coproducing solutions with intended beneficiaries.

### Recommendation 3: build stronger and sustainable localised communities

In balancing offline and online ways of interacting, there is opportunity to capitalise on the potential for more localised, coherent communities due to scaled-down travel, homeworking and local focus that will ideally continue after restrictions end. There are potential economic benefits after the pandemic, such as increased trade as home workers use local resources (eg, coffee shops), but also relational benefits from stronger relationships around the orbit of the home and neighbourhood. Experience from previous crises shows that community volunteer efforts generated early on will wane over time in the absence of deliberate work to maintain them. Adequately funded partnerships between local government, third sector and community groups are required to sustain community assets that began as a direct response to the pandemic. Such partnerships could work to secure green spaces and indoor (non-commercial) meeting spaces that promote community interaction. Green spaces in particular provide a triple benefit in encouraging physical activity and mental health, as well as facilitating social bonding.^50^ In building local communities, small community networks—that allow for diversity and break down ingroup/outgroup views—may be more helpful than the concept of ‘support bubbles’, which are exclusionary and less sustainable in the longer term. Rigorously designed intervention and evaluation—taking a systems approach—will be crucial in ensuring scale-up and sustainability.

The dramatic change to social interaction necessitated by efforts to control the spread of COVID-19 created stark challenges but also opportunities. Our essay highlights opportunities for learning, both to ensure the equity and humanity of physical restrictions, and to sustain the salutogenic effects of social relationships going forward. The starting point for capitalising on this learning is recognition of the disruption to relational mechanisms as a key part of the socioeconomic and health impact of the pandemic. In recovery planning, a general rule is that what is good for decreasing health inequalities (such as expanding social protection and public services and pursuing green inclusive growth strategies)^4^ will also benefit relationships and safeguard relational mechanisms for future generations. Putting this into action will require political will.

## Data Availability

Data sharing not applicable as no data sets generated and/or analysed for this study. Data sharing not applicable as no data sets generated or analysed for this essay.
